# Effect of adding growth factors during *in vitro* maturation on the developmental potentials of ewe oocytes selected by brilliant cresyl blue staining

**DOI:** 10.14202/vetworld.2021.452-456

**Published:** 2021-02-22

**Authors:** Mohamed Fathi, Amr F. Elkarmoty

**Affiliations:** 1 Department of Theriogenology, Faculty of Veterinary Medicine, Cairo University, Egypt; 2 Department of Anatomy and Embryology, Faculty of Veterinary Medicine, Cairo University, Egypt

**Keywords:** brilliant cresyl blue, embryo development, growth factors, *In vitro* fertilization, sheep

## Abstract

**Aim:**

Several factors had been concerned with the developmental competence of the sheep oocyte. This study aims to investigate the effect of adding growth factors (insulin-like growth factor 1 [IGF-1] and epidermal growth factor [EGF]) in the maturation medium of ewe oocytes selected based on brilliant cresyl blue (BCB) screening on *in vitro* maturation (IVM), fertilization, and pre-implantation embryo development.

**Materials and Methods:**

Cumulus-oocyte complexes (COCs) were obtained from the ovaries of slaughtered ewes by either aspiration or slicing techniques. COCs were *in vitro* matured in a medium containing IGF-1 and EGF (control group). For BCB screening, oocytes were stained and divided into BCB+ oocytes that matured in the same maturation conditions without adding growth factors (Group 2) or in the presence of growth factors (Group 3), and BCB− oocytes that matured in medium without growth factors (Group 4) or with growth factors (Group 5).

**Results:**

The supplementation of the maturation medium with growth factors during IVM of (BCB+) oocytes resulted in a significant increase in nuclear maturation rate (90.9%), fertilization rate (75.6%), and embryo developmental rates (60.0%, 46.7%, and 33.3% for cleavage, morula, and blastocyst, respectively).

**Conclusion:**

Culturing BCB+ oocytes in a maturation medium containing both EGF and IGF-1 showed a significant improvement in nuclear maturation, fertilization, and pre-implantation embryo development *in vitro*.

## Introduction

Assisted reproductive technologies have been reported as one of the major tools for increasing productivity in the livestock industry. In this domain, embryo transfer, *in vitro* fertilization, embryo cryopreservation, sex determination, and cloning in the small ruminant, such as sheep, had lower progress, the pregnancy rates remain low, and with high early embryonic mortalities [1]. The success of an *in vitro* fertilization protocol depends on the ability of the selected oocyte to resume meiosis and develop to blastocyst after fertilization [2]. Several factors have been concerned with the developmental competence of the oocyte, including the size of the follicle [3], hormonal stimulation [2], and maturation conditions [4]. *In vitro* maturation (IVM) of sheep oocytes followed by fertilization using capacitated spermatozoa and culturing the presumptive zygotes *in vitro* has been established in sheep [5] and also in goat [6].

Brilliant cresyl blue (BCB) has been reported as a successful non-invasive method in selecting more competent oocytes that develop to the blastocyst stage in different species as cow [7] and goats [8]. Recently, BCB screening studies on human oocytes were reported [9]. Depending on the activity of glucose-6-phosphate dehydrogenase (G6PDH) in the oocytes, BCB can differentiate between more competent oocytes that have lower G6PDH activity and showing blue coloration in their cytoplasm (BCB+) and less competent oocytes that have higher G6PDH activity, which have a reduced blue color to a colorless cytoplasm (BCB−) [9]. Media containing growth factors such as insulin-like growth factor 1 (IGF-1) or epidermal growth factor (EGF) have been reported to improve oocyte maturation, fertilization, and blastocyst development in many species, including sheep [10]. EGF triggers its receptors on the cell surface for proliferation, transphosphorylation of tyrosine residues, and resumption of meiosis in sheep oocytes [11], while IGF-1 acts as an amplifier to the action of follicle-stimulating hormone (FSH) [12].

This study aimed to investigate the developmental potentials of sheep oocytes selected using BCB with growth factors (IGF-1 and EGF) in a maturation medium on nuclear maturation, fertilization, and the pre-implantation embryo development *in vitro*.

## Materials and Methods

### Ethical approval

The study was approved by the Ethics Committee of Animal Reproduction Research Institute, Giza, Egypt.

### Study period and location

Ovaries were collected from a local slaughterhouse (Cairo, Egypt) from March 2016 to February 2017. The study was conducted at Laboratory of Animal Reproduction Research Institute, Giza, Egypt.

### Chemicals and reagents

All chemicals, media, and media constituents were purchased from Sigma-Aldrich. The practical work was done at Reproduction Research Institute, Department of Embryo Transfer and Artificial Insemination, Giza, Egypt.

### Oocytes recovery

A total of 370 ewe ovaries (ranging from 2 to 4 years old) were collected 30 min post-slaughter and placed in a thermos flask containing pre-warmed saline (30°C) fortified with 100 ug/mL of streptomycin.

The oocytes were recovered by aspiration of 2-6 mm follicles with a 20 g needle and a 3 mL syringe [13], using the slicing technique previously mentioned by El-Harairy *et al*. [14]. Oocytes with homogenous ooplasm and multiple layers of cumulus cells were selected by stereomicroscope.

### Oocytes staining by BCB

The recovered oocytes were stained using BCB as mentioned by Alm *et al*. [15]. Briefly, cumulus-oocyte complexes (COCs) were washed several times in a Dulbecco’s phosphate buffer saline modified by 0.4% bovine serum albumin, then exposed to 26 μM BCB diluted in modified PBS for 90 min at 38°C in 5% CO_2_ humidified atmosphere. COCs were transferred to the modified PBS for washing followed by examination under stereomicroscope. Oocytes with any extent of blue coloration in their cytoplasm were recorded as BCB+, while those without blue color in the cytoplasm were recorded as BCB−.

### *In vitro* oocytes maturation

The oocytes were washed 3 times in a washing medium (tissue culture medium [TCM]-199 supplemented with 10% fetal calf serum [FCS]). For ­maturation, the maturation medium (TCM-199 supplemented with 10% FCS, 0.8 mM sodium pyruvate, 2 mM L-glutamine, 10 μg FSH, 10 μg LH, 50 ng IGF-1, 50 ng EGF, and 50 μg/mL gentamycin) was the control (Group 1). Groups of 10-15 COCs were placed in a 100 μL droplet of IVM medium covered with mineral oil previously sterilized by filtration using a 50 Millipore filter membrane. The oocytes were incubated at 39°C in high humidity with 5% CO_2_ for 24 h. At the end of the maturation time, the oocytes were examined for signs of nuclear maturation after staining with the aceto-orcein stain as described by Fathi and El-Shahat [16]. For other treatment groups based on BCB screening, BCB+ oocytes were matured either in the same maturation medium without growth factors (IGF-1 and EGF) (Group 2) or with growth factors (IGF-1 and EGF) (Group 3) in the same maturation conditions. For BCB− oocytes, the oocytes were matured either in the same maturation medium without growth factors (IGF-1 and EGF) (Group 4) or with growth factors (IGF-1 and EGF) (Group 5) in the same maturation conditions.

### Sperm preparation

Fresh semen of frozen-thawed ram (aged 3 years old) was used for *in vitro* fertilization of the matured oocytes. Motile sperms were obtained using the swim-up technique as described by Fathim *et al*. [17]. Two 0.25 mL straws (previously frozen and tested at the reproduction research institute) were thawed in a water bath (38°C for 15 s) and emptied in a 15 mL centrifuge tube, modified Tyrode’s albumin lactate pyruvate (TALP) medium with Hepes modification was added to the centrifuge tube and allowed to centrifuge at 500×g. After discarding the supernatant, the sperm pellet was resuspended in a 2 mL TALP medium supplemented with 5 mm caffeine and incubated for 30 min at 39°C in a CO_2_ incubator. After swimming up, the motile sperm was picked up for oocyte insemination at a final concentration of 2**×**10^6^/mL.

### *In vitro* insemination of the matured oocytes

After 24 h of maturation, IVM oocytes were washed several times with pre-incubated fertilization medium (F-TALP) supplemented with 10% estrous ewe serum previously collected from estrous ewes.

Groups of 10-15 oocytes were coincubated with spermatozoa in a 100 μL fertilization medium for 18-24 h.

Fertilization events were determined based on the appearance of normal or enlarged sperm head in the ooplasm and presence of male and female pronuclei after staining with the aceto-orcein stain as described by Mohamed *et al*. [18].

### *In vitro* culture

After 18-24 h of coincubation of sperm with oocytes *in vitro*, the presumptive zygotes were washed several times in pre-incubated culture media (synthetic oviductal fluid medium supplemented with BSA) to remove any adherent sperm.

Groups of 5-10 zygotes were incubated in a 100 μL droplet of culture medium under mineral oil at 39°C in a CO_2_ incubator in a highly humid air until day 6 (day 0=day of insemination).

The cultured medium was refreshed by replacing half of the original medium with a similar volume of the pre-incubated fresh medium.

The cleaved embryos (2-16 cells) were evaluated 24-72 h after insemination, and development to morula and blastocyst stages was recorded further.

### Statistical analysis

Data were accessible as percentages; at least three replicates were conducted for each experimental group. Results of each group were compared with the other group by a Chi-squared test using GraphPad Prism 5 software (https://www.graphpad.com/scientific-software/prism/). Significance was recorded at p<0.05.

## Results

### Effect of adding growth factors during the IVM of BCB-selected oocytes

Growth factors supplementation during the IVM of BCB+ oocytes showed a significant (p<0.05) increase in maturation rate (90.9%) than BCB+ oocytes without growth factors (74.4%) and the control (77.8%). The lowest values were obtained in BCB− oocytes either matured in the presence or absence of growth factors (68.6% and 59.8%, respectively), as shown in Table-1.

**Table-1:** Effect of growth factors addition during IVM on maturation rate of BCB selected oocytes.

Groups	No. of oocytes	Oocytes maturation

		No, (%)
Control	81	63 (77.8)^b^
bcb+oocytes	82	61 (74.4)^b^
BCB+oocytes with growth factors (IGF 1 and EGF)	98	89 (90.9)^a^
BCB oocytes	92	55 (59.8)^c^
Bcb oocytes with growth factors (IGF 1 and EGF)	86	59 (68.6)^bc^

Values with different superscripts in the same column are significantly different at (p<0.05), IVM=*In vitro* maturation, BCB=Brilliant cresyl blue, IGF=Insulin like growth factor, EGF=Epidermal growth factor

### Effect of growth factor supplementation during the IVM of BCB-selected oocytes on fertilization rates

The fertilization rate of positively selected oocytes based on the BCB screening with the addition of growth factors during the IVM showed the highest value (75.6%) than the other groups. There was no significant difference observed between BCB+ oocytes without growth factors during the IVM and the control group in terms of fertilization (60.7% and 62.3%, respectively). The lowest values of fertilization rates were found in BCB− oocytes either with or without growth factors (51.3% and 48.7%, respectively), as shown in Table-2.

**Table-2:** Effect of addition of growth factors during IVM of BCB selected oocytes on the fertilization rate.

Groups	No. of inseminated oocytes	Fertilization rates

		No, (%)
Control	77	48 (62.3)^b^
Bcb+oocytes	84	51 (60.7)^b^
Bcb+with growth factors (IGF 1 and EGF)	82	62 (75.6)^a^
Bcb oocytes	78	38 (48.7)^c^
Bcb oocytes with growth factors (IGF 1 and EGF)	80	41 (51.3)^c^

Values with different superscripts in the same column are significantly different at (p<0.05). IVM=*In vitro* maturation, BCB=Brilliant cresyl blue, IGF=Insulin like growth factor, EGF=Epidermal growth factor

### Effect of growth factors supplementation during the IVM of BCB-selected oocytes on pre-implantation embryo development

The percentages of cleavage, morula, and blastocyst obtained from the IVM of BCB+ oocytes with growth factors followed by fertilization (60.0%, 46.7%, and 33.3%) were significantly (p<0.05) higher than those obtained from the IVM of BCB+ oocytes without growth factors and the control (41.9%, 29.1%, and 18.6%; 44.3%, 31.8%, and 20.5%, respectively). Interestingly, all of the previously mentioned values were significantly (p<0.05) higher than those recorded using BCB− oocytes with or without growth factors addition (31.9%, 19.1%, and 9.6%; 28.4%, 16.8%, and 7.4%, respectively), as shown in Table-3.

**Table-3:** Effect of growth factors addition during IVM of BCB selected oocytes on the percentages of cleavage, morula and blastocyst development.

Groups	No. of inseminated oocytes	Cleavage (2 16 cell stage)	Morula	Blastocyst

		No. (%)	No. (%)	No. (%)
Control	88	39 (44.3)^b^	28 (31.8)^b^	18 (20.5)^b^
Bcb+oocytes	86	36 (41.9)^b^	25 (29.1)^b^	16 (18.6)^b^
Bcb+oocytes with growth factors (IGF 1 and EGF)	105	63 (60.0)^a^	49 (46.7)^a^	35 (33.3)^a^
Bcb oocytes	95	27 (28.4)^c^	16 (16.8)^c^	7 (7.4)^c^
Bcb oocytes with growth factors (IGF 1 and EGF)	94	30 (31.9)^c^	18 (19.1)^c^	9 (9.6)^c^

Values with different superscripts in the same column are significantly different at (p<0.05). IVM=*In vitro* maturation, BCB=Brilliant cresyl blue, IGF=Insulin like growth factor, EGF=Epidermal growth factor

## Discussion

Oocyte selection based on the BCB screening has been previously established in other species at different concentrations, such as 26 μm for cow [19] and goat [20], while 13 μm of BCB was the concentration of choice in porcine oocytes [21].

Adding growth factors to the maturation medium was demonstrated by Guler *et al*. [11] who found that EGF promotes cumulus expansion and chromatin condensation, while IGF1 stimulates the proliferation of granulosa cells due to the presence of IGF-1 receptors on its plasma membrane.

The current work revealed that the supplementation of the maturation medium of BCB+ oocytes with both EGF and IGF-1 resulted in a significant increase in the maturation, fertilization, and pre-implantation embryo developmental rates than the other tested groups.

Similarly, Wang *et al*. [22] demonstrated that the BCB screening test allowed a selection of large oocytes with higher mitochondrial activity, allowing higher maturation, fertilization, and a significant increase in the percentage of embryo developmental rates in sheep. Silva *et al*. [23] added that the developmental potentials of the bovine oocytes selected based ion BCB staining showed a significant increase in the percentage of morula and blastocyst developments than those selected based on morphological characteristics. On the contrary, BCB− oocytes were less competent due to the delayed replication of the mitochondrial DNA; therefore, the oocytes failed to behave normally [24].

Concerning with growth factors supplementation, Dhanraj and Purohit [25] found that adding 50 ng of EGF to the maturation medium of goat oocytes resulted in significantly (p<0.05) higher maturation rate (52.35%) than the control (34.07%) and also significantly (p<0.05) increased the fertilization rate (28.27%) than the control (9.83%). Nearly similar to our results, Dinesh and Purohit [26] found that using both EGF and IGF-1 on the maturation medium of buffalo oocytes resulted in a higher maturation rate (83.52%) than using IGF-1 alone (67.08%) and EGF alone (63.64%) and significantly (p<0.05) higher than the control (46.30%), and a higher fertilization rate (48.62%) than IGF-1 alone (36.36%) and significantly (p<0.05) higher than the control (15.00%). Moreover, Pawshe *et al*. [27] reported that the percentage of cleavage and blastocyst development using both EGF and IGF-1 (66.0% and 21.2%) was significantly (p<0.05) higher than the control group (38.5% and 14.8%). Lonergan *et al.*, Harvey and Kaye, Lee and Fukui, and Simmen *et al*. [28-31] suggested that the increased cleavage, morula, and blastocyst development may be attributed to the positive effect of both EGF and IGF-1 on oocytes maturation. Similarly, Down *et al*. [32] reported that EGF helps in breaking the germinal vesicle and enhances the maturation rate of denuded oocytes to the MII stage.

## Conclusion

The current study demonstrated the usefulness of the supplementation of a maturation medium with both 50 ng of EGF and IGF-1 during the IVM of sheep oocytes selected by BCB staining on the proportion of *in vitro* nuclear maturation, fertilization, and subsequent sheep embryos development pre-implantation.

## Authors’ Contributions

MF: Designed the paper, data collection, made the practical part, analyzed the data, and wrote the manuscript. AFE: Designed the paper, collection of ovaries, follow-up embryonic developmental stages, and revision of the paper. Both authors have read and approved the final manuscript.
